# Fluorescence Properties of ZnOQDs-GO-g-C_3_N_4_ Nanocomposites

**DOI:** 10.3390/mi14040711

**Published:** 2023-03-23

**Authors:** Tianze Liu, Lei Wang, Ruxue Jiang, Yashi Tang, Yuxin He, Changze Sun, Yuguang Lv, Shuang Liu

**Affiliations:** 1 College of Clinical Medicine, Jiamusi University, Jiamusi 154007, China; 2 College of Pharmacy, Jiamusi University, Jiamusi 154007, China; 3 School of Mechanical Engineering, Jiamusi University, Jiamusi 154007, China; 4 College of Basic Medicine, Jiamusi University, Jiamusi 154007, China

**Keywords:** nanocomposites, fluorescence probe, metal ion, fluorescence properties

## Abstract

In this paper, the fluorescence properties of ZnOQD-GO-g-C_3_N_4_ composite materials (ZCGQDs) were studied. Firstly, the addition of a silane coupling agent (APTES) in the synthesis process was explored, and it was found that the addition of 0.04 g·mL^−1^ APTES had the largest relative fluorescence intensity and the highest quenching efficiency. The selectivity of ZCGQDs for metal ions was also investigated, and it was found that ZCGQDs showed good selectivity for Cu^2+^. ZCGQDs were optimally mixed with Cu^2+^ for 15 min. ZCGQDs also had good anti-interference capability toward Cu^2+^. There was a linear relationship between the concentration of Cu^2+^ and the fluorescence intensity of ZCGQDs in the range of 1~100 µM. The regression equation was found to be F_0_/F = 0.9687 + 0.12343C. The detection limit of Cu^2+^ was about 1.74 μM. The quenching mechanism was also analyzed.

## 1. Introduction

ZnO is an eco-friendly oxide semiconductor, and compared with traditional CdSe or CdTe quantum dots, it is an inexpensive luminescent material, which makes it more attractive in practical applications [1,2,3]. Over the past few years, a variety of physical and chemical synthesis techniques have been developed to synthesize ZnO QDS. Various chemical methods have been developed to synthesize ZnO nanocrystals, and many research groups have used the sol–gel method to improve the process of obtaining ZnO quantum dots with different sizes and fluorescence characteristics [4]. ZnO quantum dots have been widely used in the photoelectric field [5,6,7], biomedical field [8] energy electrochemical field [9,10,11,12], fluorescence imaging [13], gas sensing [14] selective detection [15], and other fields.

Graphene oxide (GO) is a carbon-based nanomaterial widely used for adsorption and catalytic degradation due to its high specific surface area and tunable structure [16]. It is also a promising nanomaterial for a wide range of applications, including but not limited to energy storage [17,18], electrical sensors [19,20], and antimicrobial activity [21,22]. GO exhibits excellent performance in photocatalytic activity due to its high electron mobility, good electrical conductivity and chemical stability. GO can be involved in the synthesis of various composite materials, such as nanoparticles, polymers, and novel metal–organic frame-derived materials, which can be used in separation [23], catalysis [24], electrochemical devices [25], and coatings [26].

In recent decades, a type of non-metallic semiconductor graphitic carbon nitride (g-C_3_N_4_) has attracted great attention among researchers worldwide because it has good photocatalytic activity under visible light irradiation and shows great potential in various environmental remediation applications. As a visible light-responsive non-metallic organic semiconductor, it has been widely used in sewage treatment [27], CO_2_ reduction [28,29], hydrogen production from water photolysis [30,31] energy conversion [32,33], and other fields.

Yang [34] et al. established a highly efficient and stable Al^3+^ fluorescent probe using the fluorescent “on” mode of zinc sulfide crystal composite zinc oxide quantum dots (ZnS/ZnOQDs). The as-synthesized ZnS/ZnOQDs exhibited fluorescence enhancement for Al, confirming the availability of fluorescence sensing probes.

Copper is a toxic heavy metal element. Cu^2+^ is involved in many reaction activities in the human body and plays an important role in various physiological processes such as the formation of red blood cells and the normal growth and maintenance of brain tissue, kidney, heart, and other organs of the body [35]. However, the content of Cu^2+^ is also a double-edged sword, and the excess or deficiency of Cu^2+^ can lead to the disorder of cellular homeostasis and damage the central nervous system. The abnormal accumulation of Cu^2+^ will lead to the appearance of some human diseases, including Alzheimer’s disease, Parkinson’s disease, Menkes disease, and Wilson’s disease. If Cu^2+^ is deficient in the human body, gastrointestinal diseases and abnormal bone growth will occur [36].

Copper accumulation in the body will produce certain harm [37]. In addition, heavy metal ions affect the activities of proteins and enzymes in the human body and accumulate in some organs, resulting in chronic poisoning [38]. Heavy metal pollution seriously endangers human health, which has raised great concern in the world. Therefore, it is of great significance and urgency to develop a rapid and sensitive detection method for heavy metal ions for environmental and public safety.

The traditional detection methods of heavy metal ions include electrochemical analysis [39,40], atomic absorption spectroscopy [41], atomic emission spectroscopy, inductively coupled plasma mass spectrometry [42], and surface-enhanced Raman spectroscopy [43,44], but these methods have the problems of cumbersome detection steps, high instrument costs, difficulty in real-time detection, and low sensitivity. Their application in the field of heavy metal ion detection is greatly restricted [45]. Compared with traditional detection methods, fluorescence analysis methods have the advantages of high resolution, good sensitivity and rapid response, and have strong potential for application. In most cases, the fluorescence intensity of fluorescent probes is related to metal ions, so it is relatively easy to qualitatively and quantitatively analyze heavy metal ions by measuring the fluorescence intensity [46]. Therefore, it is of great significance to develop fluorescent probes that can selectively detect important metal ions such as copper.

In this paper, the selectivity of ZnOQD-GO-g-C_3_N_4_ composite materials (ZCGQDs) for metal ions and the mixing time, anti-interference ability, linear relationship, and fluorescence quenching mechanism of ZCGQDs composite materials and the target metal ion Cu^2+^ were analyzed, which provides a new idea for the research of zinc oxide quantum dots.

## 2. Experimental

### 2.1. Experimental Reagents

The inorganic compounds containing Al^3+^, Ba^2+^, Ca^2+^, Cd^2+^, Co^2+^, Cr^3+^, Cu^2+^, Fe^2+^, Fe^3+^, Hg^2+^, Mn^2+^, Mg^2+^, Ni^2+^, Pb^2+^. AlCl_3_, BaCl_2_, CaCl_2_, CdCl_2_·25H_2_O, CoCl_2_·6H_2_O, CuCl_2_·2H_2_O, CrCl_3_·6H_2_O, FeCl_2_·4H_2_O, Fe(NO_3_)_3_ 9H_2_O, HgCl_2_, Mn(NO_3_)_2_·4H_2_O, Mg(NO_3_)_2_·6H_2_O, NiCl_2_·6H_2_O, and Pb(NO_3_)_2_ were purchased from Sinopharm Chemical Reagent Co., Ltd. (Huai’an, China). 

### 2.2. Metal Ion Detection

#### 2.2.1. Selective Detection of Metal Ions Based on ZCGQDs

- The inorganic compounds containing Al^3+^, Ba^2+^, Ca^2+^, Cd^2+^, Co^2+^, Cr^3+^, Cu^2+^, Fe^2+^, Fe^3+^, Hg^2+^, Mn^2+^, Mg^2+^, Ni^2+^, and Pb^2+^ were used as metal ion sources, dissolved in deionized water to prepare 50 mM solutions of different metal ions;
- A pipette was used to accurately pipette 0.005 mL of different metal ion solutions of the same concentration configured in step a;
- Then, 4.995 mL of the prepared ZCGQDs composite sample was taken;
- Various metal ions were added to the ZCGQDs composite sample to obtain a 50 μM colloidal solution of different metal ions and left for 20 min;
- A quartz cuvette was used, with a diameter of 1 cm and transparent on all sides, and the different metal ion colloid solutions in step d were added;
- The mixed colloidal solutions were scanned to obtain their fluorescence spectra and intensity values. To reduce systematic errors, each mixed colloidal solution was scanned three times, and the average was calculated as the final intensity result.

The fluorescence intensities of different mixed colloidal solutions were compared to determine the selectivity of the fluorescent probes for metal ions.

#### 2.2.2. Determination of the Linear Relationship between ZCGQDs and Target Metal Ion Cu^2+^

In order to explore the selectivity of the prepared ZCGQDs complexes to Cu^2+^, the relationship between Cu^2+^ concentration and the change in the fluorescence intensity of the ZCGQDs complexes was investigated.

Experimental process:

- Cu^2+^ was derived from the CuCl_2_·2H_2_O inorganic compound, which was dissolved in deionized water, and several solutions with different concentrations of Cu^2+^ were prepared using the stepwise dilution method;
- A pipette was used to accurately pipette 0.005 mL of Cu^2+^ solutions at the different concentrations configured in step a;
- Then, 4.995 mL samples of the ZCGQDs composites were taken;
- Different concentrations of Cu^2+^ solution were added to the ZCGQDs composite sample, mixed and shaken well to obtain a colloidal solution, which was left for 15 min;
- A quartz cuvette was used, with a diameter of 1 cm and transparent on all sides, and the colloidal solutions with different concentrations of Cu^2+^ in step d were added;
- The mixed colloidal solutions were scanned to obtain their fluorescence spectra and intensity values. In order to reduce systematic errors, each mixed colloidal solution was scanned three times, and the average intensity value was used as the representative.

### 2.3. Experimental Optimization

The addition of metal ions can cause the fluorescence intensity of ZCGQDs complexes to weaken, resulting in a fluorescence quenching phenomenon. Among these metal ions, the fluorescence intensity of the solution changed most significantly after the addition of Cu^2+^.Using this principle, the ZCGQDs complexes were used as fluorescent probes to detect Cu^2+^. However, it should be noted that there were differences in experimental settings, and different quenching efficiencies were obtained. We optimized the experiment in two aspects: (a) the addition of different concentrations of APTES in the preparation of ZCGQDs complexes; (b) the mixing of ZCGQDs complexes and Cu^2+^ at different times.

## 3. Results and Discussion

### 3.1. Effect of APTES Concentration Used to Synthesize ZCGQDs

In order to explore the effect of the addition of different concentrations of APTES on the quenching efficiency during the preparation of synthetic ZCGQDs complexes, the experiments were conducted as follows: (a) During the preparation of the ZCGQDs composites, 0.02 g·mL^−1^, 0.04 g·mL^−1^, 0.06 g·mL^−1^, and 0.08 g·mL^−1^ of a silane coupling agent (APTES) were added; (b) the ZCGQDs complexes were scanned with a fluorescence spectrophotometer to obtain their fluorescence spectra and fluorescence peak intensity values; (c)The fluorescence intensity changes of the solutions before and after the addition of Cu^2+^ were measured, and the fluorescence intensity values were labeled as F_0_ and F, respectively; the corresponding fluorescence quenching rates were represented by F_0_/F. To reduce systematic errors, the above procedure was repeated three times and the average fluorescence intensity values were calculated.

**Figure 1:**
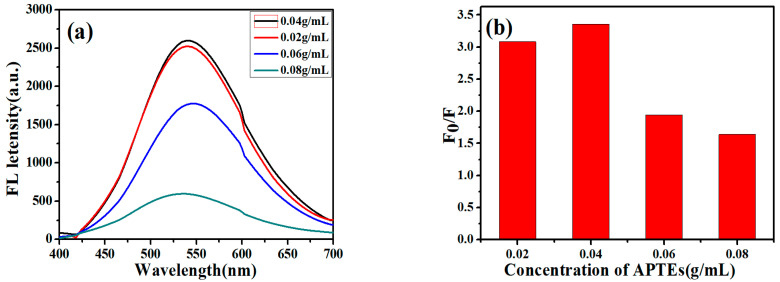
(**a**) Fluorescence emission spectra of ZCGQDs; (**b**) relative fluorescence intensity with the addition of 20 µM Cu^2+^ to ZCGQDs.

From Figure 1a, it can be concluded that with the increase in the APTES concentration, the fluorescence intensity of the ZCGQDs complexes increased. However, when the APTES concentration increased to a certain extent, the fluorescence intensity decreased instead of increasing. The reason may be that the luminescence of ZnOQDs in the visible light range is due to the deep-level emission caused by lattice defects. This is mainly caused by two kinds of defects: (1) Starting from the lower part of the conduction band, the electrons gradually leap toward the O_Zn_ and O_i_ deep body defects; (2) starting from the deep body defect V_O_, the electrons gradually leap to the upper end of the valence band. Therefore, there should be a certain concentration range and an optimum value. As shown in Figure 1a, which illustrates the change in the APTES concentration and the fluorescence intensity of the ZCGQDs complexes, the wavelength blue shift occurred when the APTES concentration exceeded 0.02 g·mL^−1^, which indicates that the appropriate increase in the APTES concentration was beneficial to the ZCGQDs complexes. The surface quickly formed a coating, which in turn reduced the agglomeration of the ZCGQDs complexes. By adding 0.04 g·mL^−1^ APTES, the fluorescence intensity was the highest.

Figure 1b shows the relative fluorescence intensity values after the addition of equal amounts of Cu^2+^ (20 μM) to the complexes formed by different concentrations of APTES and ZCGQDs. With the increase in the APTES concentration, the agglomeration degree of the ZCGQDs complexes weakened, the relative fluorescence intensity increased, and the quenching efficiency increased. However, when the APTES concentration increased to a certain extent, the relative fluorescence intensity did not increase but decreased. The excessive increase in the APTES concentration caused the coating layer of the ZCGQDs complexes to be too thick, thus destroying the fluorescence emission of the quantum dots caused by surface defects, decreasing the relative fluorescence intensity, and reducing the quenching efficiency. Notably, 0.04 g·mL^−1^ APTES had the highest relative fluorescence intensity and the highest quenching efficiency. Therefore, the concentration of 0.04 g·mL^−1^ APTES was selected to synthesize the ZCGQDs complexes for subsequent experimental analysis.

### 3.2. Effect of Mixing Time of ZCGQDs and Cu^2+^

**Figure 2:**
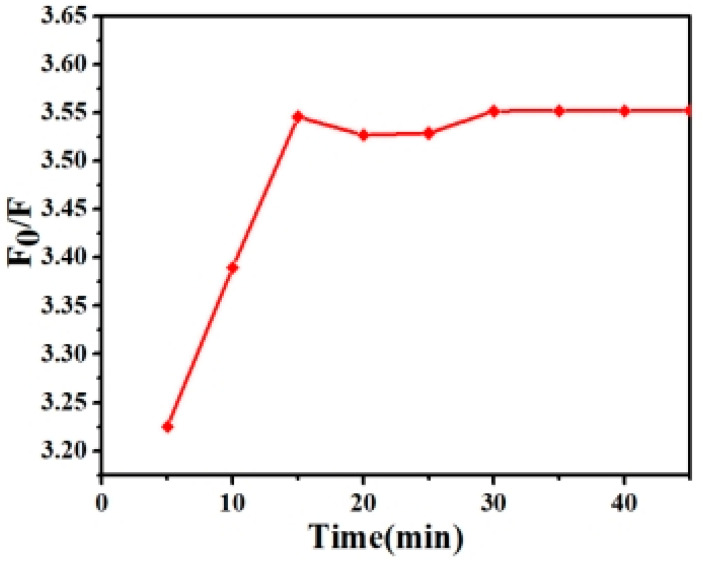
The plot of relative fluorescence intensity versus time.

As shown in Figure 2, nine time points were selected from 5 to 45 min to determine the extent of the fluorescence quenching of Cu^2+^ and ZCGQDs complexes at different mixing times [47,48]. In the range of 5–15 min, the quenching efficiency enhanced with increasing time and remained basically stable after 15 min. The highest efficiency was reached at 15 min. In order to improve the fluorescence properties of the ZCGQDs complexes in subsequent experiments, a fixed mixing time of 15 min was selected.

### 3.3. Selectivity of ZCGQDs to Metal Ions

Equal concentrations of various metal ions were added to the ZCGQDs composite sample to obtain a 50 μM colloidal solution of different metal ions and allowed to stand for 15 min [49]. The changes in the fluorescence intensity of the ZCGQDs complexes before and after the addition of Cu^2+^ were measured. The fluorescence intensity values were marked as F_0_ and F, and the corresponding fluorescence quenching rate was expressed as F_0_/F. In order to reduce the systematic error, the above process was repeated three times, and the fluorescence average intensity values were calculated.

**Figure 3:**
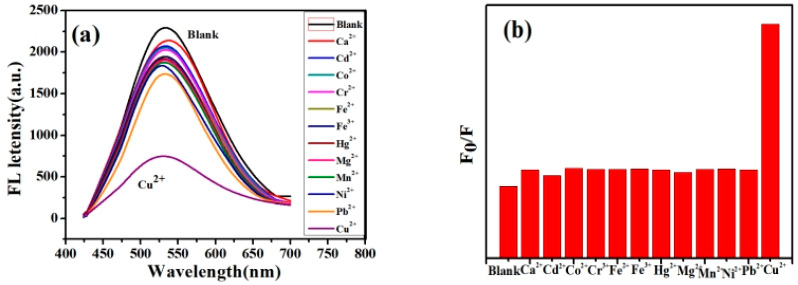
(**a**) Fluorescence spectra of the different heavy metal ions added; (**b**) relative fluorescence intensity of the different heavy metal ions added.

Figure 3a,b show the effects of different metal ions on the fluorescence intensity of the ZCGQDs complexes under the same concentration conditions. It was found that Cu^2+^ resulted in a marked fluorescence quenching phenomenon on the ZCGQDs complexes, and the as-prepared ZCGQDs complexes exhibited good selectivity toward Cu^2+^.

### 3.4. Anti-Interference Performance of ZCGQDs on Cu^2+^

**Figure 4:**
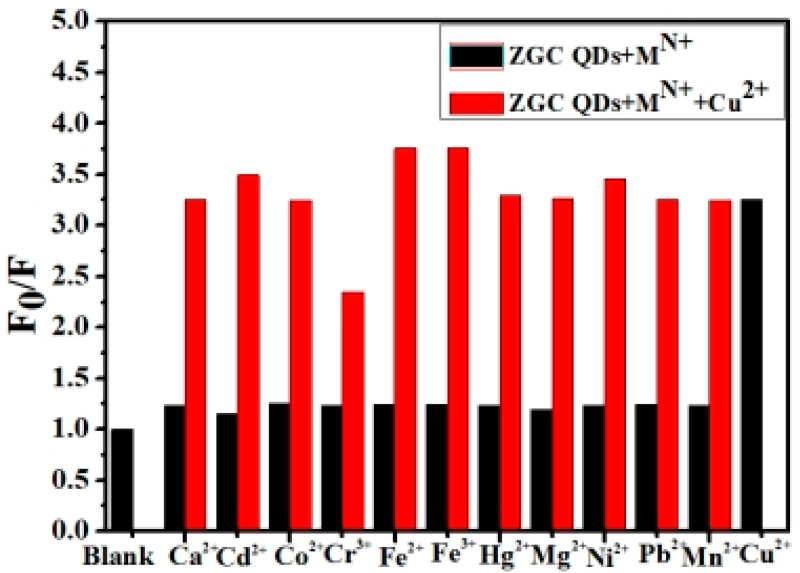
Relative fluorescence intensity of ZCGQDs in the coexistence of Cu^2+^ with other metal ions.

Different metal ions of equal concentration were prepared and added to ZCGQDs composite samples, and the mixtures were shaken to obtain the colloidal solutions of the different metal ions. Then, Cu^2+^ was added to the solutions and shaken well, and the mixtures were left to stand for 15 min to test their anti-interference performance [47]. The changes in the fluorescence intensity of the ZCGQDs complexes before and after the addition of Cu^2+^ were measured. The fluorescence intensity values were marked as F_0_ and F, and the relative fluorescence intensity (marked as F_0_/F) was used to represent the corresponding fluorescence quenching rate. In order to reduce the systematic error, the above process was repeated three times, and the average fluorescence intensity values were calculated. As shown in Figure 4, Cu^2+^ still exhibited strong fluorescence quenching under the same concentration of different metals, and the fluorescence quenching efficiency did not significantly change compared with Cu^2+^ without interference, which indicates that the prepared ZCGQDs composites had good anti-interference capability toward Cu^2+^.

### 3.5. Linear Relationship between ZCGQDs and Cu^2+^ Concentration

The stepwise dilution method was used to obtain the Cu^2+^ solutions of different concentrations, which were added to the ZCGQDs composite samples, mixed, and shaken to obtain the colloidal solutions of copper ions, and the solutions were allowed to stand for 10 min [48,49]. The changes in the fluorescence intensity of the ZCGQDs complexes before and after the addition of Cu^2+^ were measured. The fluorescence intensity values were marked as F_0_ and F, and the relative fluorescence intensity (marked as F_0_/F) was used to represent the corresponding fluorescence quenching rate.

**Figure 5:**
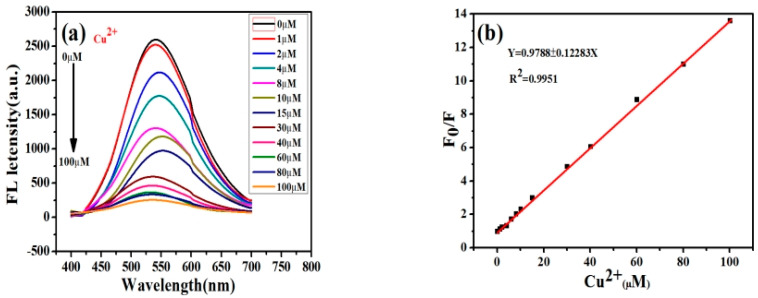
(**a**) Fluorescence spectra of different concentrations of Cu^2+^; (**b**) fitted curve of the linear relationship.

From Figure 5a, it can be concluded that the fluorescence intensity of the ZCGQDs complexes decreased sequentially with the increase in Cu^2+^ concentration. This shows a linear relationship between the concentration of Cu^2+^ and the fluorescence intensity of ZCGQDs complexes. As seen in Figure 5b, the linear regression equation for the concentration of Cu^2+^ between 1 and 100 μM is F_0_/F = 0.9788 + 0.12283C (R^2^ = 0.9951), where C represents the concentration of Cu^2+^ in the solution and R^2^ represents the correlation constant. Using this equation, the limit of detection for Cu^2+^ was obtained, which was about 1.74 μM.

### 3.6. Analysis of Fluorescence Quenching Mechanism of ZCGQDs

Copper ions had a marked fluorescence quenching effect on the ZCGQDs complexes, and there was a strong linear relationship between the Cu^2+^ concentration and the relative fluorescence intensity of the ZCGQDs complexes.

**Figure 6:**
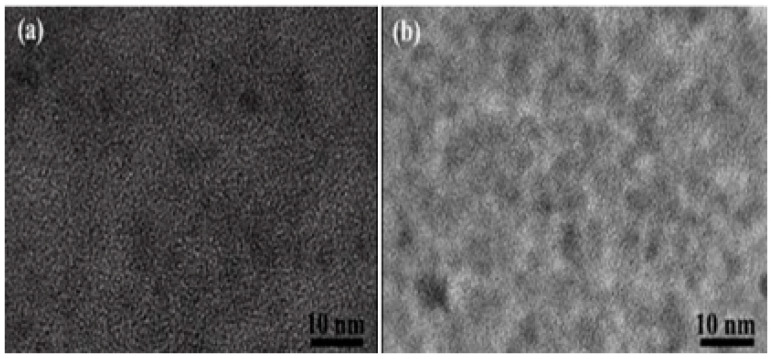
(**a**) HRTEM image of ZCGQDs; (**b**) HRTEM image of ZCGQDs after Cu^2+^ was added.

Figure 6a,b shows the particle dispersion maps before and after the addition of Cu^2+^ to the ZCGQDs composite solution. A comparison was made using transmission electron microscopy and aggregation phenomenon was observed in the ZCGQDs composite, with a consequent weakening of the fluorescence intensity. Thus, the aggregation phenomenon in the ZCGQDs composite induced fluorescence quenching (AIQ) of the ZCGQDs complex, which was caused by Cu^2+^.

The addition of metal ions can lead to fluorescence quenching of quantum dots. There are two mechanisms for this phenomenon. One is dynamic quenching, that is, after the quenching agent is added, it can carry out frequent energy transfer between the fluorescent material and reduce the fluorescence lifetime of the material. The other is static quenching, in which the two are mixed together to form a corresponding non-fluorescent complex that shows exactly the specific absorption peak when exposed to UV light.

**Figure 7:**
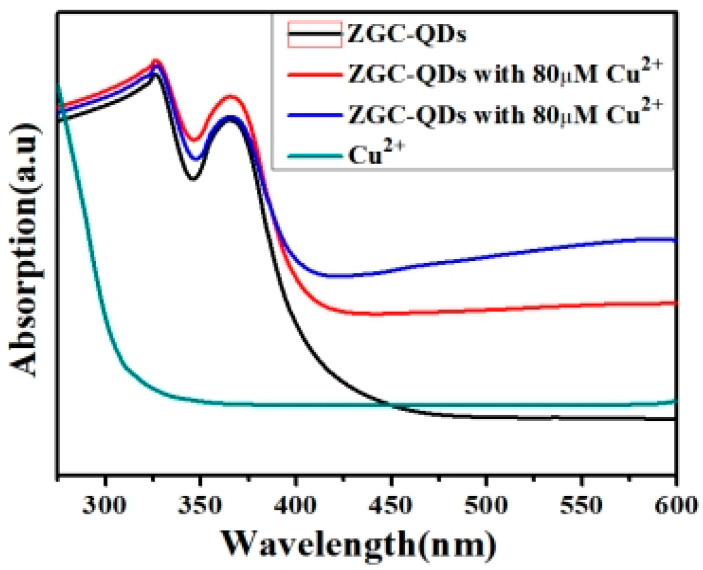
UV–vis absorption spectra of mixed colloids.

As shown in Figure 7, after the different concentrations of Cu^2+^ were added to the ZCGQDs solutions, their ultraviolet absorption spectra were plotted, and no obvious difference was observed. This indicates that the sol system formed a new non-fluorescent complex, and the reason for the fluorescence quenching of ZCGQDs complexes by Cu^2+^ was the dynamic quenching caused by energy transfer.

## 4. Conclusions

In this paper, the fluorescence performance of ZCGQDs was analyzed, and it was found that the addition of metal ions would cause the fluorescence intensity of ZCGQDs complexes to weaken, resulting in fluorescence quenching. The fluorescence quenching phenomenon produced by Cu^2+^ was the most obvious. Using this principle, the ZCGQDs complexes were used as fluorescent probes to detect Cu^2+^. The mixing time of 15 min makes the most obvious fluorescence burst of Cu^2+^ on ZCGQDs with good anti-interference performance. There was a linear relationship between ZCGQDs and Cu^2+^ in the range of 1–100 µM, with F_0_/F = 0.9687 + 0.12343C, so the detection limit of Cu^2+^ was obtained, which was about 1.74 µm. In addition, the characterization of the materials using transmission electron microscopy and UV spectroscopy proved that Cu^2+^ induced the fluorescence quenching of ZCGQDs complexes, which involved the induced aggregation quenching and dynamic quenching.

## Data Availability

The data is unavailable due to privacy or ethical restrictions.
